# Crucial Role of Nucleic Acid Sensing via Endosomal Toll-Like Receptors for the Defense of *Streptococcus pyogenes in vitro* and *in vivo*

**DOI:** 10.3389/fimmu.2019.00198

**Published:** 2019-02-21

**Authors:** Anna Hafner, Ulrike Kolbe, Isabel Freund, Virginia Castiglia, Pavel Kovarik, Tanja Poth, Franziska Herster, Markus A. Weigand, Alexander N. R. Weber, Alexander H. Dalpke, Tatjana Eigenbrod

**Affiliations:** ^1^Department of Infectious Diseases, Medical Microbiology and Hygiene, Heidelberg University Hospital, Heidelberg, Germany; ^2^Department of Anesthesiology, Heidelberg University Hospital, Heidelberg, Germany; ^3^Max F. Perutz Laboratories, University of Vienna, Vienna Biocenter, Vienna, Austria; ^4^Center for Model System and Comparative Pathology, Institute of Pathology, Heidelberg University Hospital, Heidelberg, Germany; ^5^Department of Immunology, Interfaculty Institute of Cell Biology, Eberhard-Karls-University, Tübingen, Germany; ^6^Institute of Medical Microbiology and Hygiene, Technical University Dresden, Dresden, Germany

**Keywords:** *Streptococcus pyogenes*, bacterial RNA, nucleic acids, TLR13, UNC93B1, innate immunity, skin infection

## Abstract

*Streptococcus pyogenes* is a major human pathogen causing a variety of diseases ranging from common pharyngitis to life-threatening soft tissue infections and sepsis. Microbial nucleic acids, especially bacterial RNA, have recently been recognized as a major group of pathogen-associated molecular patterns (PAMPs) involved in the detection of *Streptococcus pyogenes* via endosomal Toll-like receptors (TLRs) *in vitro*. However, the individual contribution and cooperation between TLRs as well as cell-type and strain specific differences in dependency on nucleic acid detection during *S. pyogenes* infection *in vitro* have not been clarified in detail. Moreover, the role of particularly bacterial RNA for the defense of *S. pyogenes* infection *in vivo* remains poorly defined. In this study, we report that in all investigated innate immune cells involved in the resolution of bacterial infections, including murine macrophages, dendritic cells and neutrophils, recognition of *S. pyogenes* strain ATCC12344 is almost completely dependent on nucleic acid sensing via endosomal TLRs at lower MOIs, whereas at higher MOIs, detection via TLR2 plays an additional, yet redundant role. We further demonstrate that different *S. pyogenes* strains display a considerable inter-strain variability with respect to their nucleic acid dependent recognition. Moreover, TLR13-dependent recognition of *S. pyogenes* RNA is largely non-redundant in bone marrow-derived macrophages (BMDMs), but less relevant in neutrophils and bone marrow-derived myeloid dendritic cells (BMDCs) for the induction of an innate immune response *in vitro*. *In vivo*, we show that a loss of nucleic acid sensing blunts early recognition of *S. pyogenes*, leading to a reduced local containment of the bacterial infection with subsequent pronounced systemic inflammation at later time points. Thus, our results argue for a crucial role of nucleic acid sensing via endosomal TLRs in defense of *S. pyogenes* infection both *in vitro* and *in vivo*.

## Introduction

In case of infection, the innate immune system is essential for the rapid initiation of an immune response against invading pathogens. To this end, innate immune cells employ a broad repertoire of pattern recognition receptors (PRR) recognizing highly conserved pathogen-associated molecular patterns (PAMPs) in order to activate an antimicrobial defense program. Among all PRRs, Toll-like receptors (TLRs) constitute one of the best characterized families. Upon ligand binding and subsequent activation, TLRs recruit the adaptor proteins MyD88 or TRIF, which then trigger an intracellular signaling cascade through activation of NF-κB or, in case of TRIF, the IFN regulatory factor (IRF) pathway, culminating in the induction of genes encoding proinflammatory cytokines such as IL-6, TNFα, or type I IFNs, respectively (1–3). Surface localized TLRs mainly respond to microbial cell wall components such as lipids and lipoproteins, with TLR2 recognizing lipopeptides and lipoteichoic acid (LTA) from gram-positive bacteria (4). Besides cell wall components, microbial nucleic acids represent a major group of immune stimulatory PAMPs that are detected by TLRs localized in endosomes, therefore requiring internalization to the endosomes before initiating TLR signaling. Nucleic acid sensing TLRs include TLR3 and TLR7, stimulated by viral double-stranded (dsRNA) and single-stranded RNA (ssRNA), respectively, whereas TLR9 has been identified as receptor for unmethylated CpG motifs abundant in bacterial but not in mammalian genomes (5–7). Since bacterial RNA (bRNA) has been discovered as an important activator of innate immune responses, the pathways by which bRNA mediates innate immune stimulation have been extensively investigated (8–10). Only recently, endosomal TLR13 was identified as a receptor for bRNA in murine myeloid dendritic cells and bone marrow-derived macrophages, exclusively sensing a highly conserved 13-nucleotide motif near the active site of bacterial 23S rRNA (11, 12). Even single point mutations within this short sequence motif abolish recognition by TLR13 (11, 12), thereby emphasizing its high sequence specificity, a feature unique among all innate immune receptors. In humans, however, TLR13 is not existing, albeit human monocytes respond well to bRNA stimulation with secretion of proinflammatory cytokines (9, 12). It has recently been shown that TLR8 serves as functional human TLR13 counterpart, although TLR8 does not show the strict sequence specificity observed for TLR13 (13). Endosomal TLRs require UNC93B1, an endoplasmic reticulum transmembrane protein mediating the delivery from the endoplasmic reticulum to endosomes (14, 15). Consequently, functional defects in UNC93B1 abolish endosomal TLR signaling (14, 15).

TLR signaling has been demonstrated to play a crucial role in detection of *S. pyogenes* (16), a gram-positive, major human pathogen known to cause a variety of diseases ranging from mild pharyngitis to life-threatening skin and soft tissue infections (17). Despite the availability of effective antibiotic therapy, invasive *S. pyogenes* infections such as necrotizing fasciitis require a more aggressive treatment, including surgery and supportive care in an intensive care unit (18), nonetheless resulting in approximately 163,000 deaths annually worldwide (17). Therefore, during the past years, efforts were made to decipher the different TLR pathways involved in functional recognition of *S. pyogenes*. While it is well-established that MyD88 is indispensable for launching a robust host immune response against *S. pyogenes in vitro* and *in vivo* (19, 20), the relative contribution of one single or several TLRs remains unresolved (16, 21). Recent *in vitro* studies highlighted the role of bacterial nucleic acid recognition via endosomal TLRs and especially of bRNA recognition via TLR13 for activation of innate immune cells during *S. pyogenes* infection (13, 21). However, the individual contribution and cooperation between TLRs as well as cell-type specific differences in sensing nucleic acids under variable bacterial burden are incompletely understood. In particular, endosomal TLR engagement upon *S. pyogenes* challenge in neutrophils, recognized as the most abundant immune cell population at bacterial infection sites (22, 23), has not yet been investigated. Moreover, to date, more than 200 *S. pyogenes* strains with large inter-strain variability in their genome content have been characterized (24, 25), and recent research indicates that different strains of *S. pyogenes* display a great heterogeneity in both the acute adaptive and innate immune responses they induce (26, 27). Previously published studies in the field of nucleic acid recognition in *S. pyogenes* infection were mostly based on experiments with only one single strain, and a possible inter-strain variability with respect to the dependency on nucleic acid detection in innate immune cells has not been explicitly addressed. Importantly, also the relevance of nucleic acid sensing for the defense of *S. pyogenes in vivo* remains incompletely understood (21, 28).

In the current study, we demonstrate that nucleic acid sensing plays a non-redundant role in initiating an innate immune response upon infection with *S. pyogenes in vitro* for infections with moderate bacterial load. The relative dependency on nucleic acid sensing and on sensing of *S. pyogenes* RNA via TLR13 in particular is critically influenced by the bacterial strain, multiplicity of infection (MOI) and the type of immune cell investigated. We provide evidence that in an *in vivo* model of subcutaneous *S. pyogenes* infection, the loss of endosomal TLR signaling blunts early recognition and containment of *S. pyogenes*, thus resulting in increased systemic inflammation.

## Materials and Methods

### Reagents

DMEM and RPMI 1640 containing stable glutamine were obtained from Biochrom (Berlin, Germany). FCS was purchased from Gibco/Invitrogen (Karlsruhe, Germany); Lipofectamine 2000 and TRIzol from Invitrogen Life Technologies (Darmstadt, Germany); Ultrapure LPS from *Salmonella minnesota* was supplied by U. Seydel (Forschungszentrum Borstel, Germany); Pam_3_CSK_4_ and R848 were purchased from Invivogen (San Diego, CA) and CpG1668 was custom synthesized by Eurofins (Luxemburg).

### Mouse Strains

Wildtype (WT), *Unc93b1 3d, Tlr23479*^−/−^, and *Tlr13*^−/−^ mice on a C57BL/6 background were housed in the animal facility of the University of Heidelberg under specific pathogen free conditions. *Tlr23479*^−/−^ and *Tlr13*^−/−^ mice have been described previously (11, 29) and were kindly provided by C. Kirschning (University Hospital Essen, Essen, Germany). *Unc93b1 3d* mice harboring a H412R missense mutation leading to a non-functional UNC93b1 protein (14) were generously provided by Prof. Dr. M. Freudenberg (Max Planck Institute of Immunobiology and Epigenetics, Freiburg, Germany). All knockout and mutant mice were backcrossed onto the C57BL/6 background for at least 8 generations.

### Murine Cell Isolation and Differentiation

Bone marrow-derived macrophages (BMDMs) and bone marrow-derived myeloid dendritic cells (BMDCs) were produced as described previously (30). For generation of BMDCs, 8 × 10^6^ bone marrow cells were seeded into a 15 cm cell culture plate in differentiation medium (RPMI 1640, supplemented with 10% FCS, 1% penicillin/streptomycin, 0.05 mM 2-mercaptoethanol as well as 1% granulocyte-macrophage colony-stimulating factor (GM-CSF)-containing supernatant from murine GM-CSF-transfected X63 cells (31, 32). Differentiated BMDCs were harvested on day 8. For generation of BMDMs, bone marrow cells were seeded into 15 cm petri dishes and grown in DMEM supplemented with 10% FCS, 1% penicillin/streptomycin and 30–50% L929-supernatant for 7 days. For isolation of neutrophils from mouse bone marrow, a negative selection of neutrophils using immunomagnetic cell separation was performed using an autoMACS pro Separator according to the manufacturer‘s instructions with the neutrophil isolation kit (Miltenyi Biotec, Bergisch Gladbach, Germany). A purity of CD11b/Ly6G double-positive cells of >95% was confirmed by FACS analysis.

### Bacterial Strains and Culture Conditions

The following microbial strains were used for experiments: *S. pyogenes* ATCC12344, *S. pyogenes* M49, *S. pyogenes* M1T1, *S. pyogenes* AP1 (the latter three strains were a kind gift from B. Kreikemeyer, University Hospital Rostock, Germany), and *E. coli* ATCC25922. *S. pyogenes* strains were grown either in Todd-Hewitt-Bouillon supplemented with 0.5% yeast extract (THB; Sigma-Aldrich, Taufkirchen, Germany) or in Brain-Heart-Infusion broth (BHI; Merck, Darmstadt, Germany) at 37°C with 5% CO_2_ without agitation in an Erlenmeyer flask. *E. coli* was grown in Luria-Bertani (LB) medium at 37°C with shaking at 200 rpm. Bacteria were harvested for infection assays within the mid-logarithmic phase growth, centrifuged at 2000 rpm for 10 min and resuspended in PBS or medium to the desired concentration.

### Preparation of Total Bacterial RNA

For isolation of bacterial RNA (bRNA), *S. aureus* ATCC25923 was grown in LB medium at 37°C with agitation until mid-logarithmic phase was reached. After lysis with lysozyme (40 mg/ml) for 30 min at 37°C, total bacterial RNA was extracted using TRIzol reagent according to the manufacturer‘s protocol. The isolated RNA was further cleaned up using the RNeasy mini kit (Qiagen, Hilden, Germany) with an integrated on-column DNA digestion step. RNA purity was confirmed by measuring the 260/230 and 260/280 absorbance ratio on a NanoDrop (Thermo Fisher Scientific, Waltham, USA).

### Infection and Stimulation of Murine Cells

Murine cells were generally infected or stimulated at a density of 1.5 × 10^5^ host immune cells/well in a 96-well flat-bottom plate. Only for ROS measurements, cell density was reduced to 5 × 10^4^ cells/well. BMDCs and neutrophils were seeded in antibiotic-free RPMI 1640 supplemented with 10% FCS, whereas BMDMs were seeded in antibiotic-free DMEM supplemented with 10% FCS. Unless indicated otherwise, cells were infected overnight with the indicated bacterial strains at various multiplicities of infection (MOIs), transfected with 2 μg/ml bRNA complexed with Lipofectamine 2000 at a ratio of 1 μl Lipofectamine 2000 per 1 μg RNA or stimulated with Pam_3_CSK_4_ (1 μg/ml), R848 (1 μg/ml) and LPS (100 ng/ml). For TLR2 blocking experiments, BMDMs were pre-treated with antibodies against mouse TLR2 (Clone T2.5, 5 μg/ml, Biolegend, San Diego, CA) or the appropriate control IgG (Clone MOPC-21, 5 μg/ml, Biolegend, San Diego, CA) 45–60 min prior to infection. 90 min after infection, extracellular bacteria were killed by adding penicillin/streptomycin.

### Cytokine Measurements

Cell-free supernatants were harvested and analyzed for levels of TNFα, IL-6, and IL-12p40 (BD Biosciences, Heidelberg, Germany) by ELISA according to the manufacturer's instructions. For evaluation of systemic cytokine levels in mice at indicated time points, whole mouse blood was transferred to microtubes with serum gel containing a clotting activator (Sarstedt, Nümbrecht, Germany), left undisturbed for 30 min and then centrifuged for 10 min at 10,000 g to harvest the serum. Serum cytokine profile was determined using either ELISA, the LEGENDplex mouse inflammation panel (Biolegend, San Diego, CA), or a Luminex-based multiplex assay (Bio-Rad, Hercules, USA) according to the manufacturer's instructions.

### Detection of ${\text{NO}}_{2}^{-}$ by Griess Assay

Under physiological conditions, NO is spontaneously oxidized to its stable and non-volatile breakdown product ${\text{NO}}_{2}^{-}$, which can be detected by Griess assay. ${\text{NO}}_{2}^{-}$ levels were quantified in cell-free culture supernatants 40 h post infection. Absorbance was measured at 550 nm with 630 nm serving as reference in a photometer (TECAN Sunrise, Männedorf, Switzerland). ${\text{NO}}_{2}^{-}$ concentrations were calculated according to the standard curve obtained by serial dilution of NaNO_2_.

### Measurements of Reactive Oxygen Species (ROS)

For detection of total Reactive oxygen species produced by BMDMs and BMDCs upon infection with *S. pyogenes*, Total Reactive Oxygen Species Assay Kit (Invitrogen, Waltham, USA) was used. Cells were seeded into sterile black-walled, clear-bottom 96-well flat-bottom plates and pre-treated with ROS Assay Stain stock solution 1 h prior to infection at a final dilution of 1:2000. 24 h post infection, total ROS production was quantified by measuring the fluorescence emission at 520 nm in a microplate reader (BMG FLUOstar Optima, Ortenberg, Germany).

### Experimental Model of Subcutaneous *S. pyogenes* Infection

The experimental model of subcutaneous *S. pyogenes* infection was performed as described previously except for the use of a different strain (33). Briefly, *S. pyogenes* strain ATCC12344, inoculated from a liquid overnight culture, was grown in Todd-Hewitt-Bouillon supplemented with 0.5% yeast extract at 37°C with 5% CO_2_ without agitation. When mid-logarithmic phase growth was reached after 8 h, bacteria were harvested by centrifugation at 2000 rpm for 10 min and resuspended in PBS. The concentration of the bacterial suspension was adjusted to 5 × 10^6^ CFU per 50 μl inoculum using Mc Farland standard, which was verified for each experiment by plating serial 10-fold dilutions of the inoculum on blood agar plates. Age-matched 9- to 11-week-old female WT, *Unc93b1* mutant, or *Tlr13*^−/−^ mice were lightly anesthetized by i.p. injections of a Ketamine/Xylazine cocktail and subcutaneously infected with 5 × 10^6^ CFU *S. pyogenes* at the left lower flank after being carefully shaved at the site of infection. 4, 8, and 24 h after infection, mice were sacrificed by CO_2_ asphyxiation. Blood was taken by left ventricle puncture and cytokine levels in the serum were analyzed. The lesion of each mouse was excised, and one half was transferred into 4% paraformaldehyde for histopathological examination, the other half was shock-frozen in liquid nitrogen for protein extraction and subsequent cytokine measurements. Mice experiments were approved by the local authorities.

### Histopathological Examination

After overnight fixation in 4% buffered paraformaldehyde, representative specimens of the skin were routinely dehydrated, embedded in paraffin, and then cut into 4 μm-thick sections. Sections were stained with H&E according to standard protocols and analyzed using a Leica DM2000 microscope (Wetzlar, Germany). Tissue sections from each animal were then graded for inflammation (score ranging from 0 = no inflammatory cell infiltration to 4 = severe inflammatory cell infiltration), bacterial abundance (score ranging from 0 = no bacteria to 3 = numerous bacteria) and for inflammation depth (score ranging from 0 to 5, with one point each for infiltration of epidermis, dermis, subcutis, superficial musculature, and deep musculature) by a blinded veterinary pathologist.

### Protein Extraction From Subcutaneous Tissue

For analysis of lesional cytokine production, total lesional protein was extracted from tissue samples using 1 mL Tissue Extraction Reagent I (Thermo Fisher Scientific, Waltham, USA) per 100 mg of tissue sample. Lesional cytokine profile was then determined using the LEGENDplex mouse inflammation panel (Biolegend, San Diego, CA) according to the manufacturer's instructions.

### Statistical Analysis

To assess statistical significance between multiple groups, the non-parametric Kruskal-Wallis test including Dunn's test to correct for multiple comparisons was used. When normal distribution could be assumed, an ANOVA test was performed, followed by Tukey's *post-hoc* test. To assess differences in presence or absence of a TLR2 blocking antibody, a linear mixed model with random effect for individual mice was used to account for individual differences between mice, followed by Tukey test. In case of comparisons between two groups, either a two-tailed Mann-Whitney-U-test or, in case of normal distribution, the two-tailed Student's *t*-test was performed. Significant differences were labeled as follows: ^*^p < 0.05; ^**^p < 0.01; ^***^p < 0.001. Analyses were performed using Graph Pad Prism 7 (Graph Pad Software, San Diego, USA) or using R version 3.3.0 with the packages nlme and multcomp.

### Ethics Statement

This study was carried out in accordance with the recommendations of the German Protection of Animals Act law and approved by the responsible regional authorities (council Karlsruhe, reference number G-98/14 and T-95/15). The protocol was likewise approved by the same council.

## Results

### Nucleic Acid Dependent Detection of *S. pyogenes* in BMDMs Is Critically Influenced by the Bacterial Strain, MOI, and Cytokine Investigated

**Figure 1:**
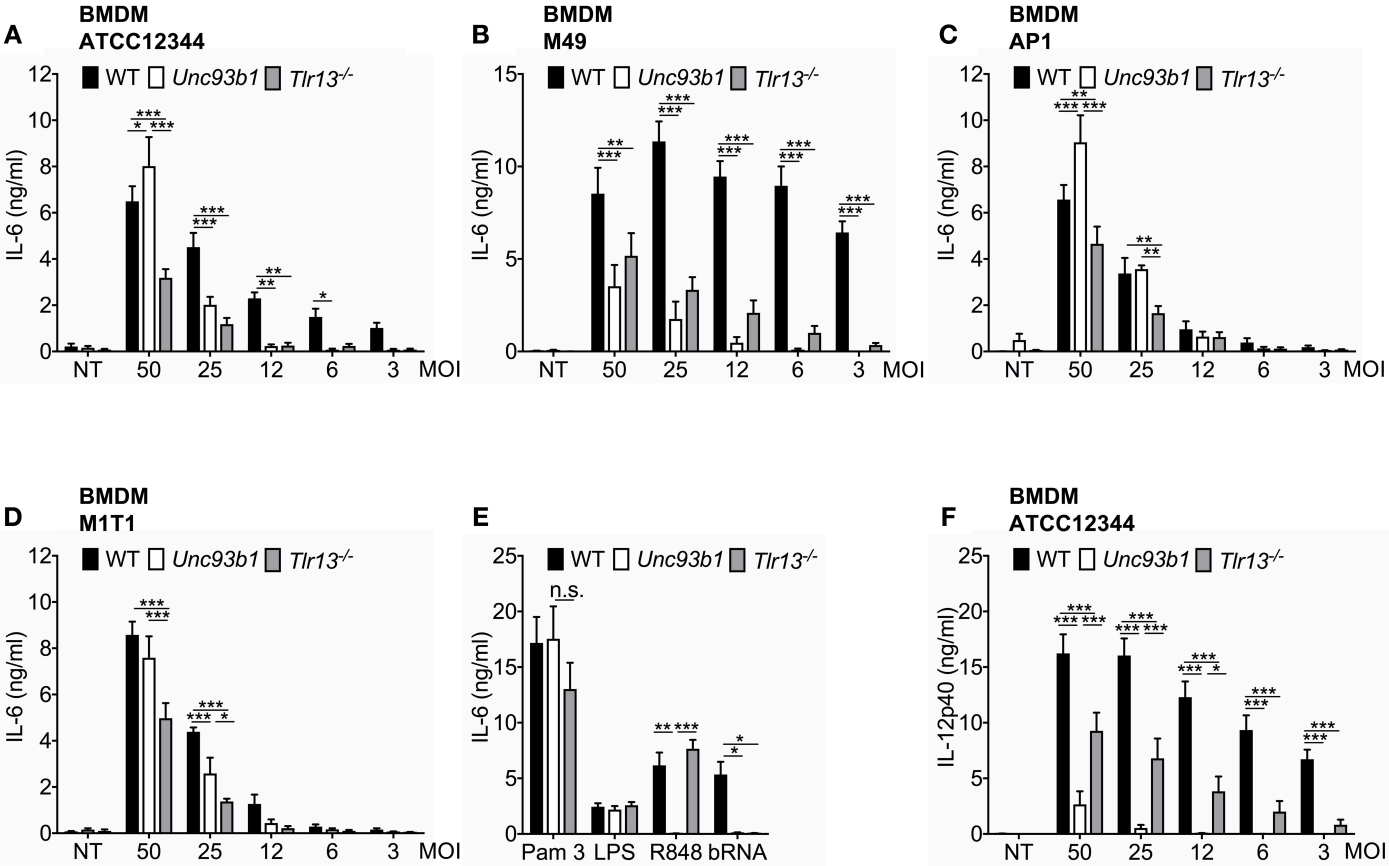
The relative contribution of nucleic acid sensing to innate immune activation upon *S. pyogenes* infection is critically influenced by bacterial strain, multiplicity of infection (MOI) and the cytokine of interest. **(A–E)** Murine bone marrow-derived macrophages (BMDMs) from wildtype (WT), *Unc93b1 3d*, and *Tlr13*^−/−^ mice were left untreated (NT) or infected over-night with live *S. pyogenes* strains ATCC12344 **(A)**, M49 **(B)**, AP1 **(C)**, or M1T1 **(D)** grown in Todd-Hewitt-Bouillon supplemented with 0.5% yeast extract at different MOIs (50, 25, 12, 6, 3). Overnight stimulation of respective BMDMs with Pam_3_CSK_4_ (Pam 3; 1 μg/ml), LPS (100 ng/ml), R848 (1 μg/ml) and bacterial RNA (bRNA; 2 μg/ml) served as controls **(E)**. IL-6 levels were analyzed in cell-free supernatants by ELISA. **(F)** As in **(A)** but IL-12p40 was analyzed by ELISA. Values represent mean data (± SEM) of three to six independent experiments. ^*^p < 0.05, ^**^p < 0.01, ^***^p < 0.001.

*Streptococcus pyogenes* has previously been reported to engage TLR2 as well as TLR13 on murine BMDMs and BMDCs, yet the relative contribution of both receptors to the overall immune response against this pathogen remains unclear and the literature is conflicting (13, 21). Moreover, limited knowledge is available about a possible inter-strain variability with regard to the relevance of nucleic acid detection for innate immune activation by *S. pyogenes*. To address these questions, BMDMs from WT, *Unc93b1 3d* mutant, and *Tlr13*^−/−^ mice were infected overnight at various MOIs with different strains of *S. pyogenes* that are commonly used in the literature. Bacteria were phagocytosed equally in WT and mutant cells (Supplementary Figure 1) and all strains triggered a dose-dependent cytokine secretion in WT BMDMs. However, in *Unc93b1* mutant, and *Tlr13*^−/−^ BMDMs production of IL-6 and TNFα upon infection with *S. pyogenes* ATCC12344 and M49 was almost abrogated at lower MOIs (Figures 1A,B and Supplementary Figure 2), indicating a non-redundant role of RNA/TLR13 recognition at low bacterial burden. Of note, nucleic acid dependent recognition was less pronounced at higher MOIs (Figures 1A,B and Supplementary Figure 2), arguing for additional recognition mechanisms that only contribute at high bacterial inoculum. Similar results were observed when bacterial uptake was blocked by cytochalasin D (Supplementary Figure 1). By contrast, *S. pyogenes* strain AP1 and M1T1 induced relevant cytokine secretion only at higher MOIs and dependency on UNC93B1 or TLR13 was less pronounced (Figures 1C,D). Together, these data reveal a significant inter-strain variability of *S. pyogenes* in terms of cytokine induction and relevance of nucleic acid sensing for mounting an innate immune response. Strikingly, culture conditions of *S. pyogenes* did not have an impact on the importance of nucleic acid detection in BMDMs (Supplementary Figure 3). Of note, enhanced IL-6 secretion in *Unc93b1* mutated vs. *Tlr13*-deficient BMDMs was observed after infection with MOI 50 (Figures 1A,C,D), potentially by compensatory upregulation of TLR2 following its stimulation to counterbalance the loss of all endosomal TLRs under high bacterial load (Supplementary Figure 4). A similar trend occurred upon stimulation with Pam_3_CSK_4_, yet did not reach statistical significance (Figure 1E). In contrast to IL-6, *S. pyogenes* ATCC12344-induced IL-12p40 production was efficiently abolished at all MOIs in *Unc93b1 3d* BMDMs, whereas the impairment of IL-12p40 production was less prominent in *Tlr13*^−/−^ cells (Figure 1F). These data suggest that endosomal TLRs other than TLR13 are likewise critical for IL-12p40 induction in BMDMs upon *S. pyogenes* infection, whereas the IL-6 response is mostly dependent on TLR13. Together, these results clearly emphasize the relevance of nucleic acid recognition, especially of bacterial RNA, for the initiation of innate immune responses against *S. pyogenes* in BMDMs, with differences in their relative contribution to overall activation according to strain, bacterial load, and individual cytokine investigated.

### Endosomal Receptors and TLR2 Mediate Innate Immune Activation by *S. pyogenes* at High MOIs, but TLR13 Plays a Dominant Role

**Figure 2:**
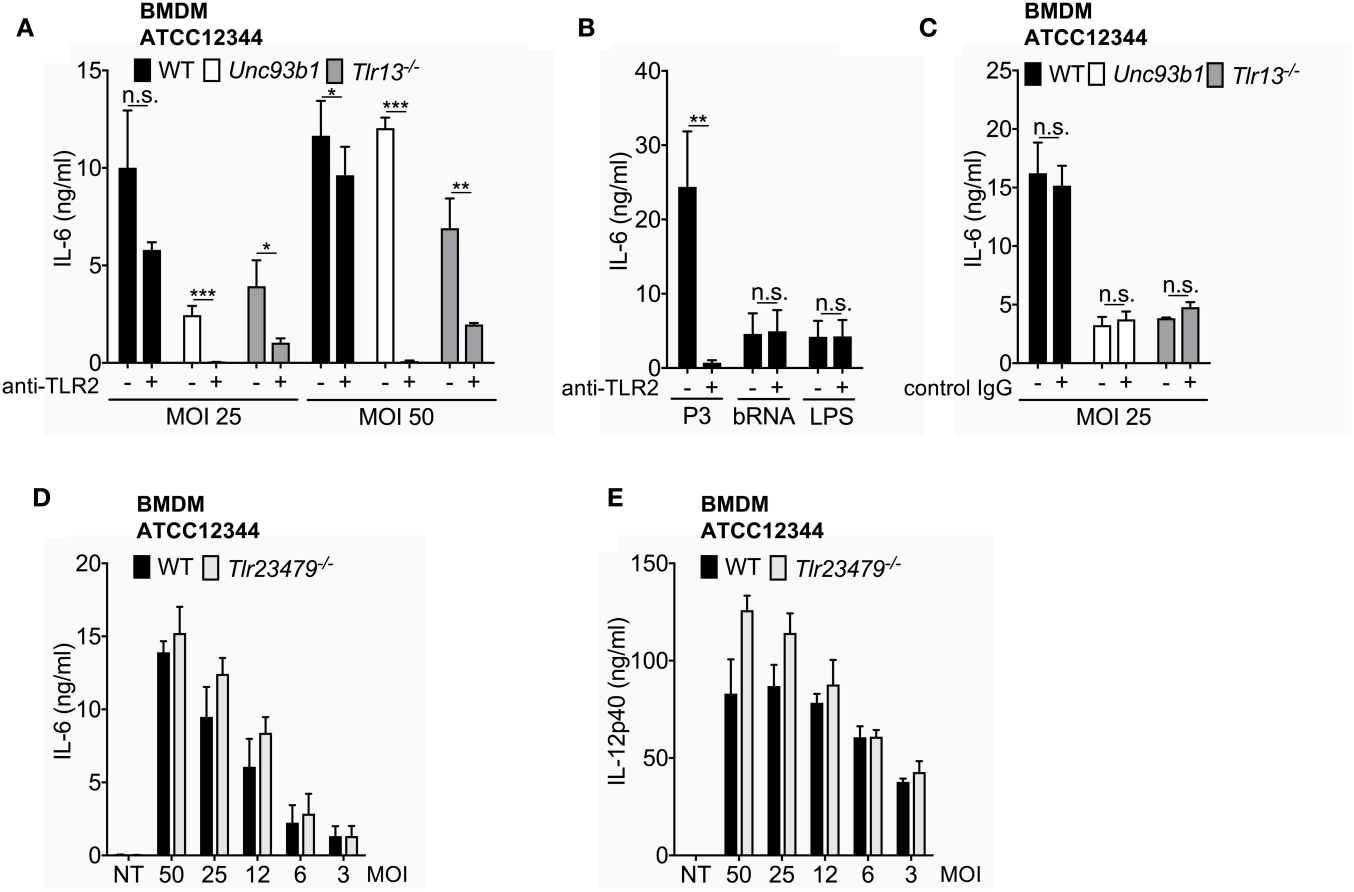
The TLR2 and TLR13 pathway are redundant in *S. pyogenes* infection at higher MOIs, whereas TLR13 signaling plays an essential, non-redundant role at lower MOIs. **(A–C)** Bone marrow-derived macrophages (BMDMs) from wildtype (WT), *Unc93b1 3d*, and *Tlr13*^−/−^ mice were infected over-night with live *S. pyogenes* strain ATCC12344 **(A)** at two MOIs (50, 25) or stimulated over-night with Pam_3_CSK_4_ (P3; 1 μg/ml), bacterial RNA (bRNA; 2 μg/ml) and LPS (100 ng/ml) **(B)** in the presence (+) or absence (–) of a TLR2 blocking **(A,B)** or a control IgG **(C)** antibody (5 μg/ml). IL-6 levels were analyzed in cell-free supernatants by ELISA. **(D,E)** BMDMs from WT and *Tlr23479*^−/−^ mice were infected overnight with live *S. pyogenes* strain ATCC12344 at different MOIs (50, 25, 12, 6, 3) or left uninfected (NT). IL-6 **(D)** or IL-12p40 **(E)** release was measured in cell-free supernatants by ELISA. Values represent mean data (± SEM) of three **(A,B)**, **(D,E)** or two **(C)** independent experiments. n.s. = not significant, ^*^p < 0.05, ^**^p < 0.01, ^***^p < 0.001.

Based on previous work by Fieber et al. (21) and the observation of an upregulation of TLR2 following its stimulation (Supplementary Figure S4), we assumed that TLR2 signaling may be responsible for the residual or even enhanced cytokine production in *Tlr13*^−/−^ and *Unc93b1* mutant BMDMs at higher MOIs. To verify this hypothesis, WT, *Unc93b1 3d*, and *Tlr13*^−/−^ BMDMs were infected with high MOIs of *S. pyogenes* ATCC12344 and AP1 (MOI 50 and MOI 25) in the presence or absence of a TLR2-blocking antibody. Inhibition of TLR2 in WT BMDMs only slightly attenuated IL-6 production upon infection with *S. pyogenes* ATCC12344, whereas cytokine production was completely abolished in *Unc93b1* mutant cells and strongly attenuated in *Tlr13*^−/−^ cells upon additional blocking of TLR2 (Figure 2A). Thus, these data suggest a redundant role of TLR2 in sensing *S. pyogenes* that becomes visible only under conditions of high bacterial load and impeded endosomal TLR signaling, with TLR13 playing the most prominent role among all nucleic acid sensing TLRs. Similar results were obtained when BMDMs were infected with the strain AP1 (Supplementary Figure 5). The presence of a TLR2-blocking antibody efficiently inhibited responses to the TLR2 ligand Pam_3_CSK_4_ without interfering with TLR13 and TLR4 signaling or the general responsiveness of BMDM as demonstrated by using isotype control antibodies (Figures 2B,C), thereby confirming efficacy and specificity of the antibody.

To further dissect the relative contribution and potential redundancies of TLR2 and endosomal TLRs with respect to *S. pyogenes* sensing, the response of cells with a combined deficiency in TLR 2, 3, 4, 7, and 9 was investigated. Intriguingly, neither IL-6 secretion nor IL-12p40 production were impaired in *Tlr23479*^−/−^ BMDMs (Figures 2D,E), indicating a redundant function of these receptors in recognition of *S. pyogenes* even at high MOIs. The same was observed when cells were infected with *S. pyogenes* AP1 strain (Supplementary Figure 5). Taken together, the results presented so far corroborate the major role of TLR13 and TLR2 in recognition of *S. pyogenes* by BMDMs. Moreover, the data identify the TLR2 and TLR13 pathway as being redundant at higher MOIs, whereas TLR13 signaling is shown to play an essential, non-redundant role at lower MOIs and to compensate completely for the loss of TLR2 and other endosomal TLRs in the defense of *S. pyogenes*. Thus, bacterial RNA represents the dominant immune stimulus for mounting an innate immune response against *S. pyogenes* in BMDMs.

### The Relative Contribution of Nucleic Acid Detection to Innate Immune Activation by *S. pyogenes* Differs Between Immune Cells

**Figure 3:**
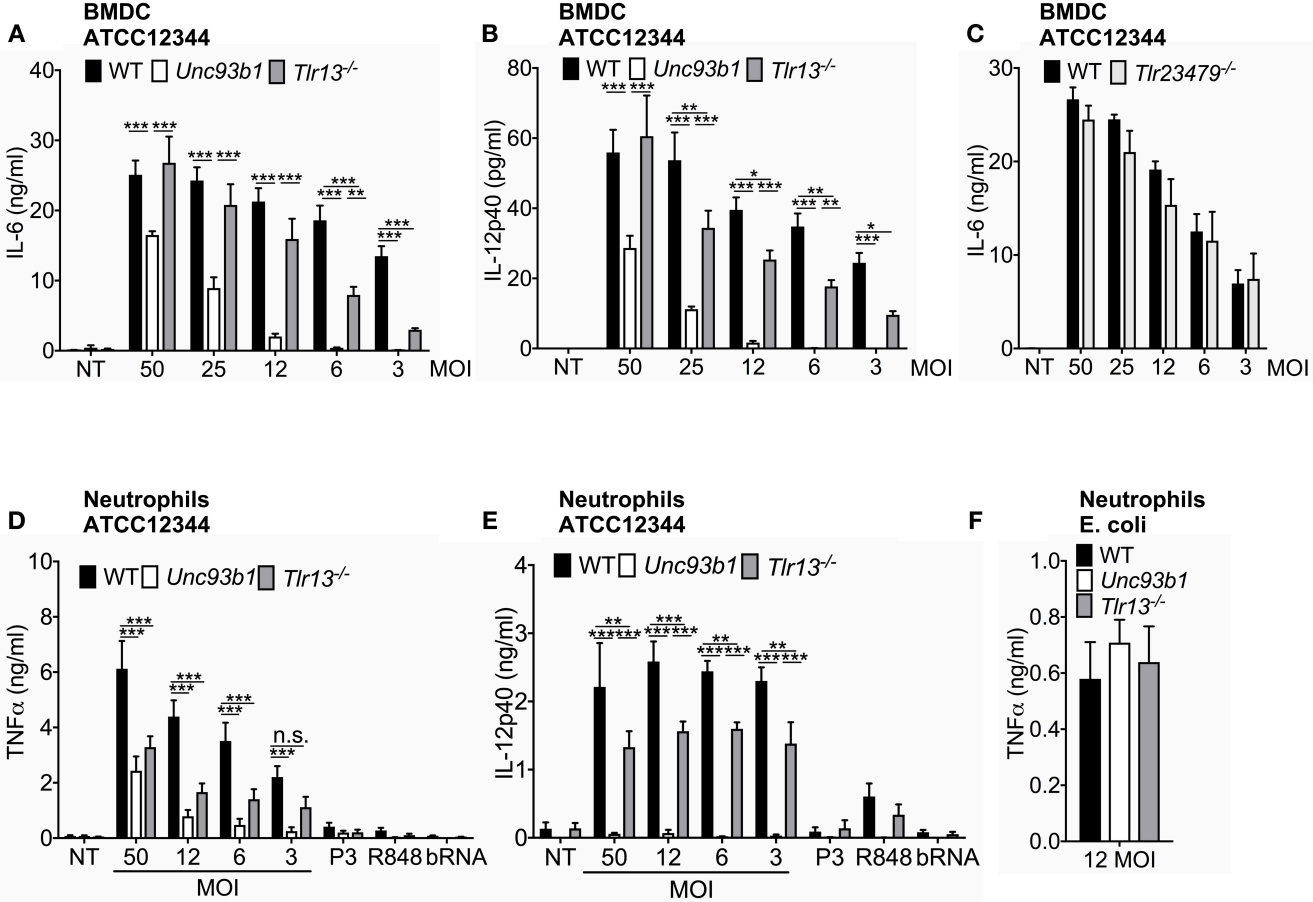
The relative contribution of nucleic acid detection to innate immune activation by *S. pyogenes* differs between immune cells. **(A,B)** Murine bone marrow-derived myeloid dendritic cells (BMDCs) from wildtype (WT), *Unc93b1 3d*, and *Tlr13*^−/−^ mice were infected overnight with live *S. pyogenes* strain ATCC12344 at different MOIs (50, 25, 12, 6, 3) or left uninfected (NT). Levels of IL-6 **(A)** and IL-12p40 **(B)** were analyzed in cell-free supernatants by ELISA. **(C)** BMDCs from WT and *Tlr23479*^−/−^ mice were infected overnight with live *S. pyogenes* strain ATCC12344 at different MOIs (50, 25, 12, 6, 3) or left uninfected (NT). **(D–F)** Murine neutrophils derived from wildtype (WT), *Unc93b1 3d*, and *Tlr13*^−/−^ mice were infected overnight with live *S. pyogenes* strain ATCC12344 at different MOIs (50, 12, 6, 3) or were stimulated with Pam_3_CSK_4_ (P3; 1 μg/ml), R848 (1 μg/ml), and bacterial RNA (bRNA; 2 μg/ml) **(D,E)** or infected with live *E. coli* strain ATCC25922 at MOI 12 **(F)**. Levels of TNFα **(D,F)** and IL-12p40 **(E)** were analyzed in cell-free supernatants by ELISA. **(A–F)** Values represent mean data (± SEM) of three to four independent experiments. n.s. = not significant, ^*^p < 0.05, ^**^p < 0.01, ^***^p < 0.001.

As host defense mechanisms against pathogens physiologically engage several cell types, we next examined whether *S. pyogenes* RNA also represents the dominant immune stimulus in cell types other than macrophages. For this purpose, we measured cytokine production in bone marrow-derived dendritic cells (BMDCs) from WT, *Unc93b1 3d* mutant, and *Tlr13*^−/−^ mice infected overnight with various MOIs of *S. pyogenes* ATCC12344. In line with the data obtained in macrophages, both IL-6 and IL-12p40 secretion in BMDCs were critically dependent on endosomal TLR signaling, especially at lower MOIs (Figures 3A,B), thus further substantiating the critical role of nucleic acid detection in cytokine induction. However, in striking contrast to macrophages, BMDCs from *Tlr13*^−/−^ mice displayed only slightly decreased cytokine levels compared to their WT counterparts (Figures 3A,B), suggesting that in BMDCs, non-redundant recognition of *S. pyogenes* RNA by TLR13 was less relevant for the induction of an immune response. Yet, BMDCs deficient in TLR2/3/4/7/9 produced comparable amounts IL-6 (Figure 3C) and IL-12p40 (Supplementary Figure 6) as WT cells, implying that in the absence of TLR2/3/4/7/9 signaling the TLR13 pathway becomes more prominent compared to WT cells.

Neutrophils represent the first line of defense in bacterial infections (22), but to date, it has not yet been investigated which TLRs are involved in recognition of *S. pyogenes* in neutrophils. We therefore further investigated the dependency of murine neutrophils on nucleic acid detection in defense of *S. pyogenes*. Infection of neutrophils isolated from WT, *Unc93b1 3d*, and *Tlr13*^−/−^ mice with *S. pyogenes* ATCC12344 revealed a similar stimulation pattern as observed in BMDCs: While in neutrophils lacking functional UNC93b1, TNFα production triggered by *S. pyogenes* was strongly impaired and IL-12p40 secretion was even completely abolished, the secretion of both cytokines was less attenuated in *Tlr13*^−/−^ cells, especially in case of IL-12p40 (Figures 3D,E). This finding clearly demonstrates the pivotal role of nucleic acid detection for mounting an immune response against *S. pyogenes* in neutrophils, whereas specific recognition of *S. pyogenes* RNA via TLR13 appears to be less prominent. In line with previously published data (34), we observed that murine neutrophils only barely respond to single TLR ligands including purified bacterial RNA (Figures 3D,E). Yet, neutrophils from all genotypes responded equally to infection with live *E. coli* which served as positive control (Figure 3F).

In conclusion, our results highlight nucleic acid detection as a prerequisite for an effective immune response in both BMDCs and neutrophils. However, in contrast to macrophages, TLR13, and the endosomal nucleic acid sensing receptors TLR3/7/9 are largely redundant in their function.

### Nucleic Acid Detection Is Crucial for Both ROS- and NO-Production Serving as Antimicrobial Agents

**Figure 4:**
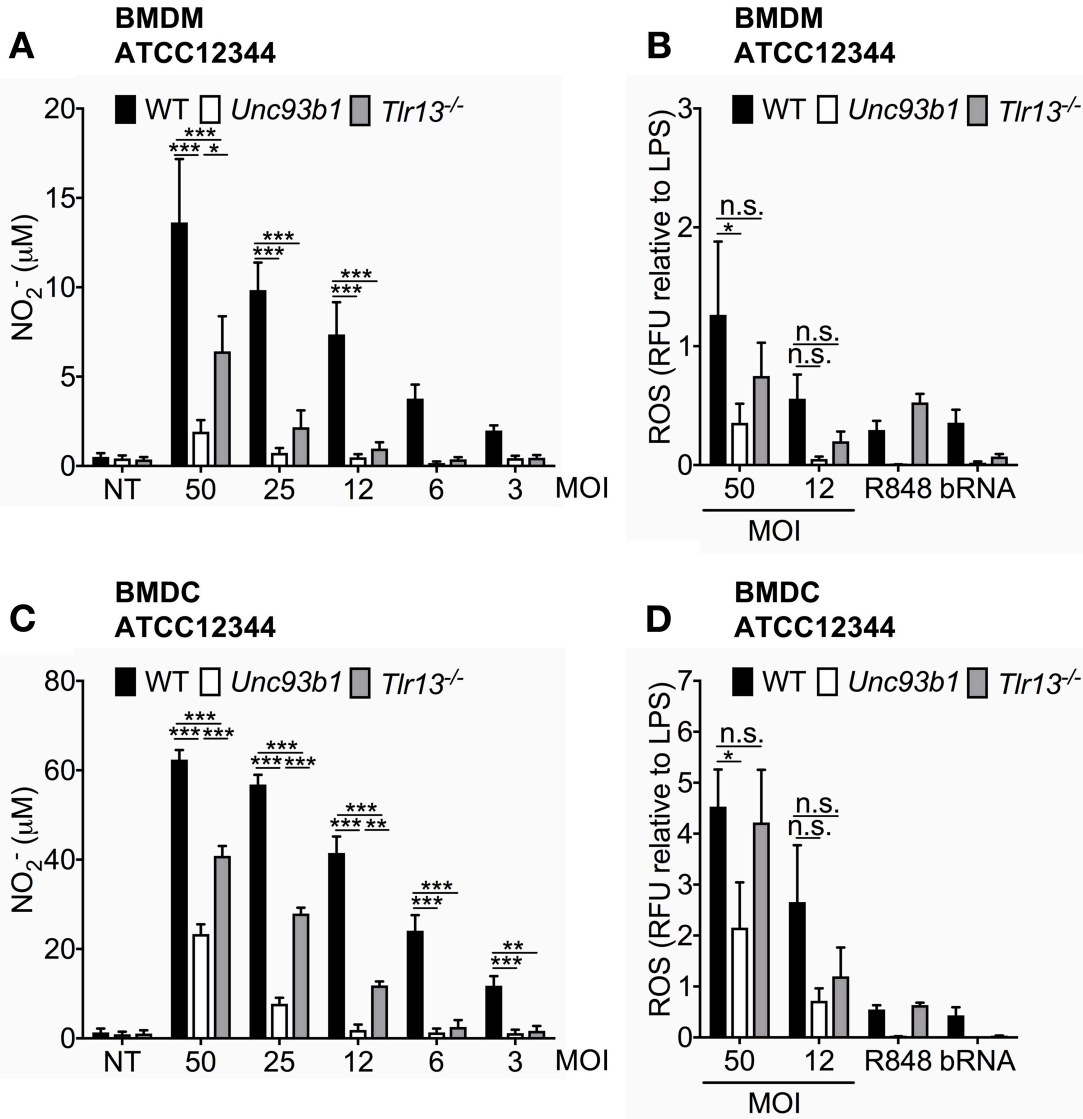
Nucleic acid detection is crucial for both ROS- and NO-production upon *S. pyogenes* infection. **(A,B)** Murine bone marrow-derived macrophages (BMDMs) as well as **(C,D)** murine bone marrow-derived myeloid dendritic cells (BMDCs) from wildtype (WT), *Unc93b1 3d*, and *Tlr13*^−/−^ mice were infected with live *S. pyogenes* strain ATCC12344 at the indicated MOIs, left untreated (NT) or stimulated with R848 (1 μg/ml) and bacterial RNA (bRNA; 2 μg/ml) as positive controls. **(A,C)** 40 h post infection, ${\text{NO}}_{2}^{-}$ levels were analyzed in cell-free supernatants by Griess assay. **(B,D)** 24 h post infection, total ROS production was quantified by measuring the fluorescence emission. Relative fluorescence units (RFU) were then normalized to LPS RFU levels. **(A–D)** Values represent mean data (± SEM) of three to five independent experiments. n.s. = not significant, ^*^p < 0.05, ^**^p < 0.01, ^***^p < 0.001.

Reactive nitrogen species (RNS) and reactive oxygen species (ROS) have been demonstrated to be a major part of an effective host response toward pathogens by exerting a direct antimicrobial activity (35–37) and thus containing the infection (38–40). However, to date, the contribution of endosomal TLR signaling to NO formation or ROS production in *S. pyogenes* infections has not yet been evaluated. We therefore assessed these parameters in BMDMs and BMDCs from WT, *Unc93b1 3d*, and *Tlr13*^−/−^ mice upon infection with *S. pyogenes* ATCC12344 *in vitro*. NO generation was strongly reduced in BMDMs lacking either functional endosomal TLRs or TLR13 alone when compared to their WT counterparts, especially at lower MOIs (Figure 4A). A similar pattern was observed for ROS production although data did not reach statistical significance for *Tlr13*^−/−^ cells (Figure 4B). Likewise, *S. pyogenes* induced NO and ROS formation was also highly dependent on nucleic acid sensing in BMDCs, as demonstrated by an impaired production of both antimicrobial agents in *Unc93b1* mutant cells (Figures 4C,D). In line with the cytokine secretion profiles (Figures 1A, 3A,B), the relative contribution of endosomal TLR signaling to NO production vanished with higher MOIs in both cell types, with recognition of *S. pyogenes* via TLR13 being less prominent in BMDCs as compared to BMDMs. Together, these findings identify nucleic acid sensing as prominent stimulus for NO formation and as contributor to ROS production in BMDMs and BMDCs upon *S. pyogenes* infection.

### Early Recognition of *S. pyogenes* ATCC12344 Is Impaired in *Unc93b1* Mutant Mice, With Subsequent Lack of Containment and Pronounced Systemic Inflammation Later During Infection

**Figure 5:**
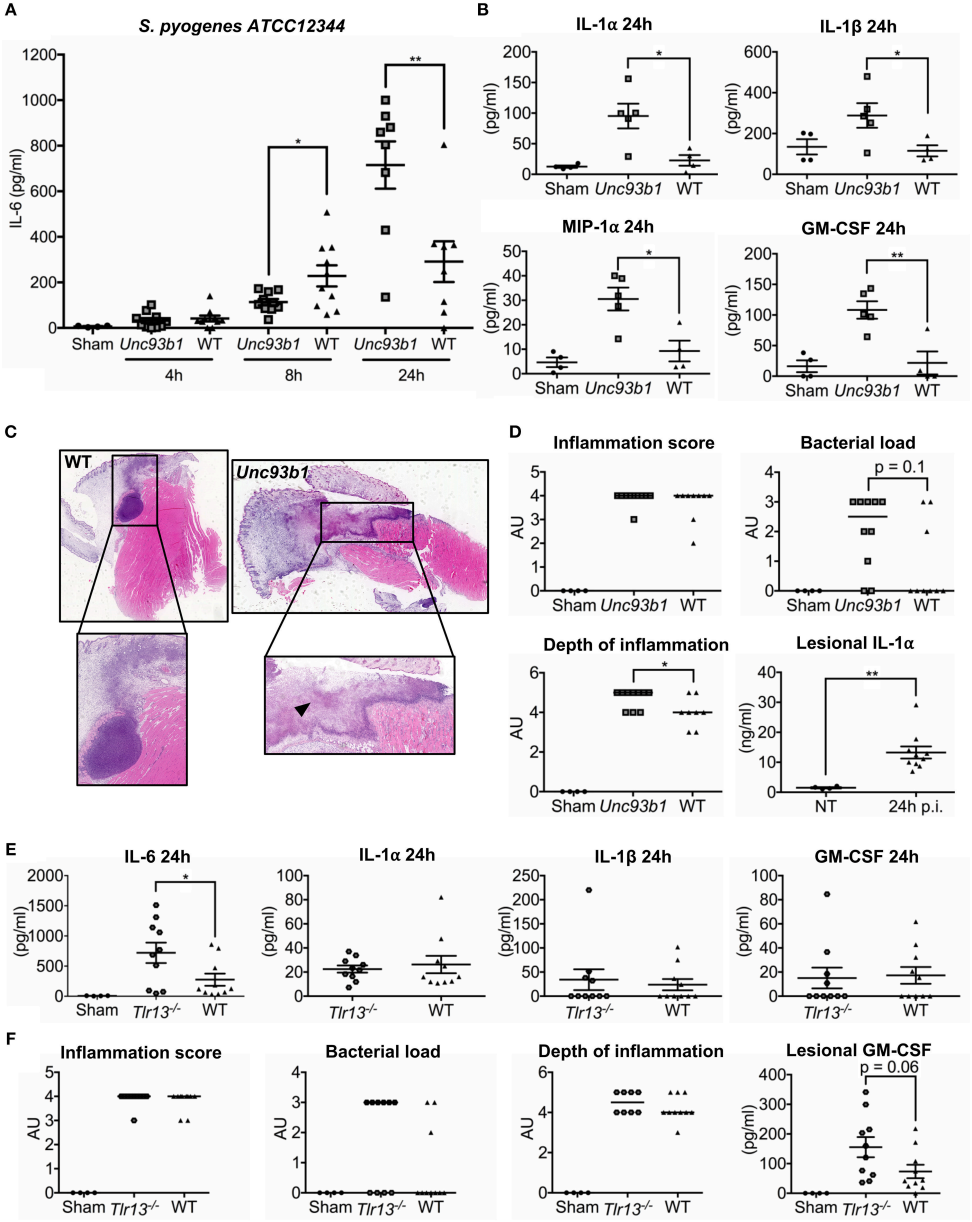
Early recognition of *S. pyogenes* ATCC12344 is impaired in *Unc93b1* mutant mice, with subsequent lack of containment and pronounced systemic inflammation later during infection. **(A–D)** *Unc93b1 3d* and WT mice were subcutaneously infected with 5 × 10^6^ CFU *S. pyogenes* strain ATCC12344 and euthanized at the indicated time points. **(A)** Systemic IL-6 levels were assessed by ELISA 4, 8, and 24 h after infection (*n* = 8–10 per genotype). Values represent mean data of two independent experiments (± SEM). **(B)** Systemic cytokine levels were analyzed by a Luminex-based multiplex assay 24 h post infection (*n* = 4–5 per genotype). Values represent mean data (± SEM). **(C,D)** H&E-stained tissue sections of the lesion from *Unc93b1 3d* and WT mice 24 h post infection were graded for inflammation, bacterial load, and depth of inflammation (*n* = 8–10 per genotype) with bars indicating the median. Representative tissue sections are shown. Arrows indicate necrotic areas **(C)**. Lesional IL-1α levels were assessed by a cytometric bead assay. Values represent mean data (± SEM) **(D)**. **(E,F)** *Tlr13*^−/−^ and WT mice were infected as in **(A)** and systemic cytokine levels were analyzed by a cytometric bead assay 24 h post infection (*n* = 10 per genotype). Values represent mean data (± SEM) of two independent experiments **(E,F)**. H&E-stained tissue sections and lesional GM-CSF levels were analyzed as in **(C)** (*n* = 10 per genotype). **(A–F)** Each data points illustrates one individual mouse. ^*^p < 0.05, ^**^p < 0.01.

Considering the importance of nucleic acid sensing for the induction of innate immune responses *in vitro*, we next set out to investigate whether nucleic acid detection is also required for defense against *S. pyogenes in vivo*. WT, *Unc93b1* mutant, and *Tlr13*^−/−^ mice were therefore subcutaneously infected with *S. pyogenes* strain ATCC12344 and sacrificed at the indicated time points. This established mouse model of soft tissue infection mimics human skin infections with *S. pyogenes* culminating in necrotizing fasciitis and sepsis (41). During the early phase of infection, *Unc93b1* mutant mice displayed significantly decreased systemic IL-6 levels compared to their WT controls (Figure 5A), indicative of an insufficient recognition of *S. pyogenes* by the innate immune cells, thus potentially favoring systemic dissemination. However, 24 h post infection, we observed overall increased systemic cytokine and chemokine levels in mice deficient in endosomal TLR signaling (Figures 5A,B), presumably due to an increased bacteremia, as demonstrated by a tendency toward higher bacterial burden in spleens from *Unc93b1* mutant mice compared to their WT counterparts (Supplementary Figure 7). At the same time, histopathological examination 24 h post infection revealed more severe lesions in *Unc93b1* mutant mice with an elevated bacterial load and more inflammatory cells invading into deeper layers of the lesions (Figures 5C,D). Notably, markedly enhanced lesional levels of the alarmin IL-1α were observed in infected mice compared to non-treated controls (Figure 5D), thus confirming the presence of a relevant tissue damage and necrosis. Together, our data demonstrate a failure in early recognition of *S. pyogenes* ATCC12344 in *Unc93b1* mutant mice, leading to a reduced containment of *S. pyogenes* in the course of infection with subsequent spread and systemic inflammation. In line with these results, *Tlr13*^−/−^ mice also displayed significantly enhanced systemic IL-6 levels compared to their WT controls 24 h after infection, albeit they did not show an overall increased systemic inflammatory response as observed in *Unc93b1* mutant mice (Figure 5E). Consistently, *Tlr13*^−/−^ mice exhibited a slightly, yet not significantly, enhanced bacterial and inflammatory burden at the site of infection, with a mildly increased local infiltration of inflammatory cells (Figure 5F). This enhanced local inflammation might be due to elevated lesional GM-CSF levels found in *Tlr13*^−/−^ mice, promoting the recruitment of inflammatory cells to the infection site (Figure 5F).

In conclusion, our results argue for a crucial contribution of nucleic acid detection via endosomal TLRs to defense against *S. pyogenes in vivo*.

## Discussion

For a long time, the immune recognition of most gram-positive bacteria has predominantly been attributed to binding of their cell wall components to TLR2. However, recent research revealed that TLR2 dependent pathways are less prominent for the recognition of streptococci (10, 16, 19, 33, 42, 43), a feature which thus appears to distinguish streptococci from other gram-positive pathogens. Although we and others have already shed light on the relevance of nucleic acids sensing via endosomal TLRs in defense of infections caused by the major human pathogen *S. pyogenes in vitro* (13, 21, 44), to date, the relative contribution of nucleic acid detection to the overall immune response against this pathogen *in vitro* and *in vivo* remains poorly defined. In the present study, we therefore applied a comprehensive approach using various experimental conditions to systematically dissect the relevance of nucleic acids for sensing of *S. pyogenes*. Our *in vitro* data now provide evidence for three main findings: (i) In all investigated innate immune cells involved in the resolution of bacterial infections, *S. pyogenes* ATCC12344 is recognized in an almost complete UNC93B1, and thus endosomal TLR, dependent manner at lower MOIs whereas at higher MOIs, recognition via TLR2 emerges but plays a redundant role; (ii) TLR13-dependent recognition of *S. pyogenes* is largely non-redundant in BMDMs, but less essential in neutrophils and especially in BMDCs; (iii) different *S. pyogenes* strains greatly vary with regard to their nucleic acid dependent recognition.

Previous studies on nucleic acid dependent recognition of *S. pyogenes* revealed conflicting data: While Fieber et al. (21) demonstrated that only a simultaneous inactivation of both the TLR2 and TLR13 pathway impaired TNFα and IL-6 production upon *S. pyogenes* infection, thus concluding that the TLR2 and TLR13 pathway were largely redundant *in vitro*, our previous studies suggested a non-redundant involvement of TLR13 in *S. pyogenes* sensing (13). The current study now provides evidence that these at first glance contradictory findings can be explained by different experimental conditions, i.e., different bacterial strains and MOIs employed by both groups (MOI 50 in Fieber et al. vs. MOI 5 in Eigenbrod et al.). Our current findings suggest that the TLR2 pathway exerts an auxiliary function, assisting the TLR13 mediated recognition of *S. pyogenes* in case of pronounced microbial challenge to ensure an efficient microbial killing.

Besides combining different experimental conditions, a major strength of this study is that various innate immune cells engaged in the resolution of bacterial infections were extensively examined, including murine neutrophils which are considered as the first line of defense in *S. pyogenes* skin infections (22). To our knowledge, we demonstrate for the first time that murine neutrophils stimulated by live *S. pyogenes* also rely on nucleic acid detection for mounting an immune response against this pathogen, thus further emphasizing the relevance of nucleic acids as PAMPs. However, murine neutrophils turned out to be less dependent on *S. pyogenes* RNA recognition, with TLR13 and the endosomal nucleic acid sensing receptors TLR3/7/9 being largely redundant in their function. Similar findings were obtained in BMDCs. Which of these endosomal TLRs plays the dominant role in case of TLR13 deficiency and whether RNA or rather DNA sensing is of relevance remains speculative. This question could only be answered using mice harboring a combined loss in TLR13 plus one or several other endosomal TLRs. The discrepancy between TLR13- und UNC93B1-dependent cytokine production was especially prominent for IL-12p40 in all investigated cell types, including macrophages, where RNA otherwise represents the dominant stimulus for cell activation. IL-12p40 is a cytokine that has previously been described to rely on prolonged NF-κB-activation to achieve sufficient gene expression, a feature primarily observed for TLR7 and TLR9 dependent pathways (45). It is therefore conceivable that IL-12p40 activating thresholds can be reached partially in TLR13 deficient cells but not in cells harboring a combined deficiency in all endosomal TLRs. Given the pivotal role of IL-12p40 as a key regulator in Th1 polarization and thus linking innate and adaptive immunity (46), the current study may shed light on the relevance of nucleic acid sensing in calling adaptive immunity into action and thus promoting immunity toward *S. pyogenes* infection. This is supported by a recent publication reporting that recognition of bacterial mRNA via TLR8 stimulates follicular helper T cell differentiation by inducing a specific cytokine profile in human monocytes upon infection with live *E. coli*, thereby promoting vaccine responses (47). In this context, *E. coli* mRNA serves as so-called “vita-PAMP” that is present only in live microbes, thus enabling innate immune cells to discriminate viable–and potentially more harmful–from dead microorganisms (48). Further investigations are necessary to elucidate which microbial components are able to link innate and adaptive immunity in *S. pyogenes* infections and to reveal the involved pathways in detail.

Former studies investigating nucleic acid recognition in *S. pyogenes* infection were mainly performed with one single *S. pyogenes* strain without explicitly addressing inter-strain variability (10, 16, 19, 21, 33, 43). However, recently published data revealed that different *S. pyogenes* strains induced heterogeneous acute adaptive immune responses, most probably due to the intra-species variability found in the genome content (27). Moreover, Dinis et al. demonstrated that also *in vitro* innate immune responses elicited by *S. pyogenes* clinical isolates varied dependent on the *emm* type in terms of type I interferon production (26). This is in line with the findings of our study, which clearly unravels an inter-strain variability with regard to both the extent of cytokine production and the dependency on nucleic acid detection in BMDMs. Thus, an observed immune response after infection with one strain might not adequately reflect the immune response signature of the entire species, which is of apparent importance for future research. A plethora of factors might contribute to the phenotype observed in our study, including variations in uptake, cell wall composition or bacterial degradation in the endolysosome and subsequent differences in accessibility of nucleic acids for TLR stimulation. Whether clinical isolates of *S. pyogenes* characterized by a pronounced genetic diversity (49) also differ in their dependency on nucleic acid detection and whether this affects patient outcomes remains to be examined.

TLR signaling has also been shown to play a crucial role in the defense of *S. pyogenes in vivo*. In the murine model of subcutaneous *S. pyogenes* infection, *MyD88*^−/−^ mice displayed an impaired survival compared to their WT counterparts (20). Moreover, Fieber et al. (21) revealed a decreased survival in the same model of *S. pyogenes* infection in *Tlr2* deficient and *Unc93b1* mutant but not in *Tlr9* deficient mice. Our data confirm the increased susceptibility of *Unc93b1* mutant mice: we show that early recognition of *S. pyogenes* is impaired in mice lacking endosomal TLR signaling, resulting in a reduced local containment of *S. pyogenes* infection with bacterial overgrowth, local hyperinflammation and consequently increased systemic cytokine levels at later time points. Overexpression of IL-1β as observed in *Unc93b1* mutant mice has previously been demonstrated to be involved in *S. pyogenes* induced hyperinflammation *in vivo* and shown to be negatively regulated by type I interferon signaling in an RNA dependent manner (28). However, given the clear-cut results obtained in *Unc93b1* mutant cells *in vitro*, the phenotype observed in *Unc93b1* mutant mice *in vivo* appears to be rather moderate, pointing toward an additional role of TLR2 *in vivo*, as suggested by Fieber et al. (21). In line with the *in vitro* data, the phenotype in *Tlr13*^−/−^ mice was even less pronounced as compared to *Unc93b1* mutant mice. In addition, a strongly reduced cytokine production has been observed in *MyD88*-deficient mice at 20 h post infection (20), while cytokine levels were significantly increased in *Unc93b1* mutant mice 24 h post infection, indicating the presence of UNC93B1-independent, but MyD88-dependent pathways. This might be partially explained by a possible interference with tissue resident cells, such as keratinocytes or Langerhans-cells, which may detect *S. pyogenes* in a nucleic acid independent manner. Another factor that might influence the phenotype independently of nucleic acid sensing pathways is toxin-mediated tissue damage. *S. pyogenes* has been reported to secrete several exotoxins that may cause cell lysis and tissue destruction (50), culminating in the formation of necrotic tissues, as also observed in our *in vivo* studies. Necrotic cells typically release a variety of danger associated molecular patterns (DAMPs), such as IL-1α identified as a major product of keratinocytes (51, 52). IL-1α has only recently emerged as an alarmin that rapidly initiates the production of pro-inflammatory mediators in a MyD88-dependent manner, thus promoting recruitment of neutrophils and macrophages to the site of infection as well as their activation independent of TLR signaling pathways (53–55). Indeed, we could show that subcutaneously infected animals displayed enhanced lesional IL-1α levels compared to uninfected controls, indicating that DAMP signaling could mask or even compensate for TLR deficiency and thus attenuate the phenotype in *Unc93b1* mutant or *Tlr13* deficient mice. The relevance of DAMP signaling in general and of IL-1α in particular should be addressed in future studies using the appropriate knockout mice.

In summary, our findings in the current study emphasize the relevance of nucleic acid sensing for the initiation of an innate immune response upon infection with *S. pyogenes in vitro* and *in vivo*.

## Data Availability

The raw data supporting the conclusions of this manuscript will be made available by the authors, without undue reservation, to any qualified researcher.

## Author Contributions

AH, UK, and IF performed experiments. AH organized the database and analyzed data, with help from UK. VC and PK provided expertise for *in vivo* experiments. FH and AW helped with neutrophil experiments. TP performed histological analysis of tissue sections. AH, MW, and AD contributed to the conceptual development of the study. AH and TE wrote the manuscript and all authors commented on and revised the manuscript. TE coordinated and supervised the entire study.

### Conflict of Interest Statement

The authors declare that the research was conducted in the absence of any commercial or financial relationships that could be construed as a potential conflict of interest.
