# Supplementary material for: Crucial Role of Nucleic Acid Sensing via Endosomal Toll-Like Receptors for the Defense of Streptococcus pyogenes in vitro and in vivo
Source: Front Immunol. 2019 Feb 21;10:198. doi: 10.3389/fimmu.2019.00198 (PMC6394247; doi:10.3389/fimmu.2019.00198)
Supplement: Supplementary file 1 [file Data_Sheet_1.docx]

Supplementary Material

Crucial role of nucleic acid sensing via endosomal Toll-like receptors for the defense of *Streptococcus pyogenes in vitro* and *in vivo*

**Anna Hafner^1,2#^, Ulrike Kolbe^1#^, Isabel Freund^1^, Virginia Castiglia^3^, Pavel Kovarik^3^, Tanja Poth^4^, Franziska Herster^5^, Markus A. Weigand^2^, Alexander N. R. Weber^5^, Alexander H.**

**Dalpke^1^, Tatjana Eigenbrod^1*^**

^1^Department of Infectious Diseases, Medical Microbiology and Hygiene, Heidelberg University Hospital, Heidelberg, Germany

^2^Department of Anesthesiology, Heidelberg University Hospital, Heidelberg, Germany

^3^Max F. Perutz Laboratories, University of Vienna, Vienna Biocenter, Vienna, Austria

^4^Center for Model System and Comparative Pathology, Institute of Pathology, Heidelberg University Hospital, Heidelberg, Germany

^5^Department of Immunology, Interfaculty Institute of Cell Biology, Eberhard-Karls-University, Tübingen, Germany.

**^#^ equal contribution**

*** Correspondence:**

Tatjana Eigenbrod: tatjana.eigenbrod@med.uni-heidelberg.de

## Supplementary Materials and Methods

**Assessment of TLR2 expression levels by quantitative real-time PCR**

For analysis of TLR2 expression levels, 5x10^6^ BMDMs from wildtype (WT), *Unc93b1 3d* and *Tlr13^-/-^* mice were seeded in 12 well plates in DMEM supplemented with 10% (v/v) heat inactivated FCS and infected with *S.* *pyogenes* ATCC12344 at MOI 50. Total RNA from cells was isolated 20h post infection using the Extract me total RNA Kit (DNA Gdansk, Poland) and reverse transcribed into cDNA using the High-Capacity cDNA Reverse Transcription Kit (Thermo Fisher Scientific, Carlsbad, CA, USA) according to the manufacturer`s instructions. Quantitative real-time PCR was performed with Fast SYBR™ Green Master Mix (Applied Biosystems, Foster City, USA) using a standard protocol. Identity of amplicons was verified by melting curve analysis, and no-reverse transcriptase and no-template controls were included. Analyses were performed in duplicates and relative expression levels of TLR2 were assessed by the comparative threshold cycle (∆C_t_) method using β-actin as housekeeping gene. Primer sequences were as follows: mouse β-actin, forward, 5´-GATGACGATATCGCTGCGCTG-3´ and reverse, 5´-GTCGACCAGAGGCATACAGG-3´; mouse TLR2, forward, 5´-GCCAAGAGGAAGCCCAAGA-3´ and reverse, 5´-AAGGGCGGGTCAGAGTTCTC-3´. All primers were custom synthesized by MWG-Biotech (Ebersberg, Germany). Fold induction (FI) was calculated by normalization to the ∆C_t_ value of WT BMDM infected with *S.* *pyogenes* ATCC12344.

## Supplementary Figures

**Supplementary Figure 1.** **Bacterial uptake is comparable in WT and mutant BMDMs and crucial for *S. pyogenes* induced cytokine secretion.**

**(A)** Murine bone marrow-derived macrophages (BMDMs) from wildtype (WT, upper panels) and *Unc93b1 3d* (lower panels) mice were infected with live, CFSE-labeled *S. pyogenes* strain ATCC12344 or left uninfected (NT). 20 hours after infection, cells were washed, fixation was performed with 4% paraformaldehyde and uptake of bacteria was analyzed by flow cytometry. Panels show representative FACS plots out of three independent experiments. Values represent mean data (± SD) of three independent experiments. **(B)** Murine bone marrow-derived macrophages (BMDMs) from wildtype (WT) and *Unc93b1 3d* mice were infected overnight with live *S. pyogenes* strain ATCC12344 at the indicated MOIs, stimulated with LPS (100 ng/ml) or left untreated (NT) in presence or absence of cytochalasin D (Cyto D, 10 μM). IL-6 levels were analyzed in cell-free supernatants by ELISA. Values represent mean data (± SEM) of four independent experiments. ** p<0.01, *** p<0.001.


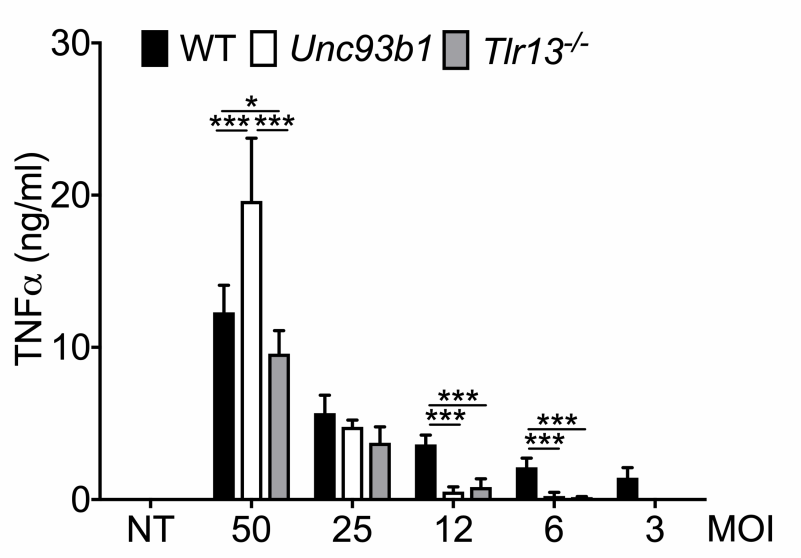


**Supplementary Figure 2.** **The relative contribution of nucleic acid sensing to innate immune activation upon *S. pyogenes* infection is critically influenced by bacterial strain, multiplicity of infection (MOI) and the cytokine of interest.**

Murine bone marrow-derived macrophages (BMDMs) from wildtype (WT), *Unc93b1 3d* and *Tlr13^-/-^* mice were left untreated (NT) or infected over-night with live *S. pyogenes* strain ATCC12344 at different MOIs (50, 25, 12, 6, 3). TNFα levels were analyzed in cell-free supernatants by ELISA. Values represent mean data (± SEM) of six independent experiments. * p<0.05, *** p<0.001.

**Supplementary Figure 3.** **Culture conditions of *S. pyogenes* do not affect the importance of nucleic acid detection in BMDMs.**

**(A-D)** Murine bone marrow-derived macrophages (BMDMs) from wildtype (WT), *Unc93b1 3d* and *Tlr13^-/-^* mice were infected overnight with live *S. pyogenes* strain ATCC12344 **(A)**, M49 **(B)**, AP1 **(C)** or M1T1 **(D)** grown in Brain Heart infusion broth instead of Todd-Hewitt-Bouillon (Fig. 1) at the indicated MOIs or left untreated (NT). IL-6 levels were analyzed in cell-free supernatants by ELISA. Values represent mean data (± SEM) of three to four independent experiments. n.s. = not significant, * p<0.05, ** p<0.01, *** p<0.001.


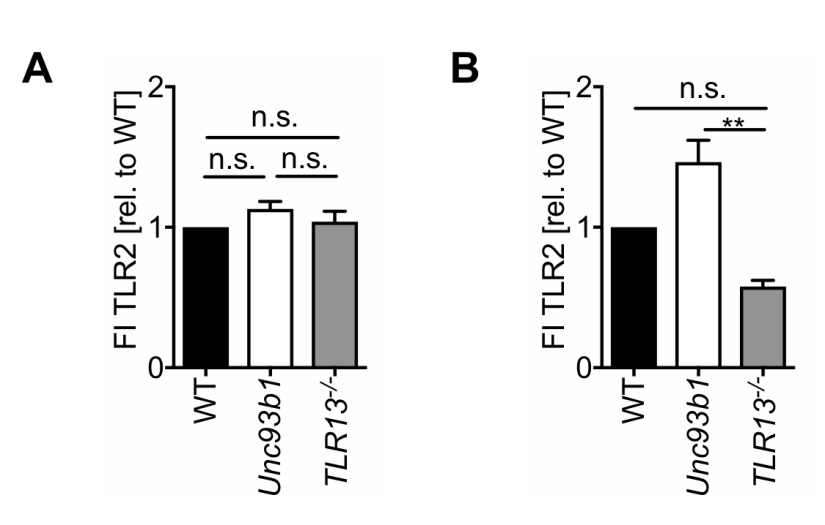


**Supplementary Figure 4.** **Upregulation of TLR2 in *Unc93b1 3d* bone marrow-derived macrophages (BMDMs) upon infection with *S. pyogenes*.**

BMDMs from wildtype (WT), *Unc93b1 3d* and *Tlr13^-/-^* mice were left untreated **(A)** or infected with *S.* *pyogenes* ATCC12344 at MOI 50 **(B)**. RNA was isolated 20 h post infection and reverse transcribed into cDNA. Expression levels of TLR2 and β-actin serving as housekeeping gene were determined by qRT-PCR. Fold induction (FI) was calculated by normalization to the ∆C_t_ value of non-treated WT BMDM **(A)** or WT BMDM infected with *S.* *pyogenes* ATCC12344**(B)**. n.s. = not significant, ** p < 0.01.

**Supplementary Figure 5.** **The TLR2 and TLR13 pathway are redundant in *S. pyogenes* AP1 infection at higher MOIs**

**(A)** Bone marrow-derived macrophages (BMDMs) from wildtype (WT), *Unc93b1 3d* and *Tlr13^-/-^* mice were infected overnight with live *S. pyogenes* strain AP1 at two MOIs (50, 25) in presence (+) or absence (-) of a TLR2 blocking antibody (5 μg/ml). **(B)** BMDMs from WT and *Tlr23479^-/-^* mice were infected overnight with live *S. pyogenes* strain AP1 at different MOIs (50, 25, 12, 6, 3) or left uninfected (NT). **(A-B)** IL-6 release was measured in cell-free supernatants by ELISA. Values represent mean data (± SEM) of three independent experiments. n.s. = not significant, * p<0.05, ** p<0.01, *** p<0.001.

**Supplementary Figure 6.** **TLR13 compensates completely for the loss of TLR2 and other endosomal TLRs in the defense of *S. pyogenes*.**

**(A)** Murine bone marrow-derived myeloid dendritic cells (BMDCs) from wildtype (WT) and *Tlr23479^-/-^* mice were infected overnight with live *S. pyogenes* strain ATCC12344 at different MOIs (50, 25, 12, 6, 3) or left uninfected (NT). IL-12p40 levels were analyzed in cell-free supernatants by ELISA. **(B)** As positive control, BMDCs from WT and *Tlr23479^-/-^* mice were stimulated overnight with Pam_3_CSK_4_ (P 3; 1 μg/ml), LPS (100 ng/ml), R848 (1 μg/ml), bacterial RNA (bRNA; 2 μg/ml) or CpG1668. IL-6 release was measured in cell-free supernatants by ELISA. Values represent mean data (± SEM) of three independent experiments.


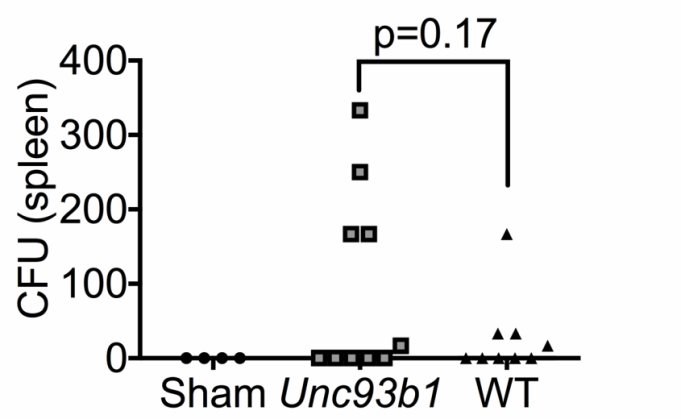


**Supplementary Figure 7.** **Higher bacterial burden in spleens from *Unc93b1* mutant mice compared to their WT counterparts.**

*Unc93b1 3d* and WT mice were subcutaneously infected with 5x10^6^ CFU *S. pyogenes* strain ATCC12344 and euthanized 24 hours post infection. Bacterial loads in murine spleens were determined. Dot plots represent a pool of two independent experiments (in total n=9-10 per genotype).
